# Genomic Analysis of the Trehalose-6-Phosphate Synthase Family Involved in Trehalose Biosynthesis and Drought Response in *Morus alba*

**DOI:** 10.3390/cimb48040356

**Published:** 2026-03-28

**Authors:** Mengting Li, Hui Gan, Xie Wang, Jiyang Wang, Leixin Deng, Hangcheng Hu, Sitong Qiao, Meng Tang, Shujie Tang, Haoran Jin, Duwei Xia, Anqi Ding

**Affiliations:** 1 College of Geography and Planning, Chengdu University of Technology, Chengdu 610059, China; lmt176163@163.com (M.L.); ganhui0918@163.com (H.G.); 18091637251@163.com (J.W.); 19940982110@163.com (L.D.); liu294266806@163.com (H.H.); qst807674168@163.com (S.Q.); 13880282879@163.com (M.T.); tequila10325@163.com (S.T.); 13866236695@163.com (H.J.); 13518310107@163.com (D.X.); 2 Research Center of National Park, Sichuan Key Research Base for Social Sciences, Chengdu 610059, China; 3 Human Geography Research Center of Qinghai-Tibet Plateau and Its Eastern Margin, Chengdu 610059, China; 4 College of Life Sciences, Northwest A&F University, Xianyang 712100, China; 5 Institute of Agricultural Resources and Environment, Sichuan Academy of Agricultural Sciences, Chengdu 610066, China; wangxiechangde@hotmail.com

**Keywords:** *Morus alba*, trehalose-6-phosphate synthase, drought stress, evolution analysis, expression pattern

## Abstract

Drought stress severely limits the growth and productivity of *Morus alba*, yet the molecular mechanisms underlying its adaptation remain poorly understood. Trehalose, an important osmoprotectant and signaling molecule, plays a key role in plant responses to abiotic stress, and its biosynthesis is primarily regulated by trehalose-6-phosphate synthase (TPS). However, the characteristics and potential functions of *TPS* genes in *M. alba* have not been systematically investigated. In this study, we identified 11 *TPS* genes (*MaTPSs*) in the *M. alba* genome and performed comprehensive analyses, including phylogenetic relationships, gene structures, conserved motifs, cis-regulatory elements, and expression profiles. Phylogenetic analysis classified *MaTPSs* into TPS I and TPS II subfamilies, with closer evolutionary relationships to *Populus trichocarpa* than to *Arabidopsis thaliana*. Promoter analysis revealed the presence of multiple stress- and hormone-responsive elements, suggesting their potential involvement in abiotic stress regulation. Physiological measurements showed that drought stress significantly increased trehalose accumulation, with a 1.6-fold increase in leaves and a 2.2-fold increase in roots. Expression profiling further demonstrated that six *MaTPS* genes were upregulated under drought stress, among which *MaTPS4*, *MaTPS9*, *MaTPS10*, and *MaTPS11* exhibited significant induction (approximately *5-*, 5-, 8-, and 10-fold, respectively). Correlation analysis further indicated that trehalose accumulation was positively associated with all upregulated *MaTPS* genes (*p* < 0.05). Taken together, these results suggest that *MaTPS* genes may be involved in drought-responsive regulation of trehalose metabolism in *M. alba*. This study provides a valuable foundation for future functional validation and the genetic improvement of drought tolerance in mulberry.

## 1. Introduction

As a non-reducing disaccharide (α-D-glucopyranosyl-α-D-glucopyranoside) trehalose is widely distributed in various biological systems such as bacteria, fungi, plants, and animals [1,2]. Trehalose effectively maintains the stability of biomacromolecules and tissues through water binding, water replacement, and vitrification [3,4]. Moreover, Trehalose is massively accumulated and functions as a protectant to preserve cell integrity and viability under cold, drought, heat and salt stresses [5]. In plants, trehalose biosynthesis occurs via the OtsA–OtsB pathway. Trehalose-6-phosphate (Tre6P), the intermediate product in the pathway catalyzed by trehalose-6-phosphate synthase (TPS) and trehalose-6-phosphate phosphatase (TPP), functions as a key signaling molecule modulating plant stress responses [6].

*TPS* and *TPP* constitute the major gene families regulating trehalose synthesis, mediating its biosynthesis and contributing to plant stress tolerance [7]. Genome-wide analyses have identified *TPS* gene families in several plants, including *Arabidopsis thaliana* [8], rice [9], wheat [10], sesame [11], *Prunus mume* [12], and *Camellia sinensis* [13], and have confirmed their widespread induction by abiotic stresses such as drought, high salinity, and low temperature. Recently, 38 *MsTPS* genes were systematically identified in *Medicago sativa* [14], and genome-wide studies in peach have highlighted potential roles of *TPS* genes in carbohydrate metabolism [15]. Functional studies on *TPP* genes in *Solanum lycopersicum* revealed that *SlTPP3* mediates responses to salt stress [16].

*TPS* genes exhibit significant divergence in structure, evolution, and expression patterns, underpinning plant adaptation to complex environments. For instance, in *Neolamarckia cadamba*, Class I *NcTPSs* contain 14–18 exons, whereas Class II genes contain 2–5 exons [17]. Similarly, in *Citrullus lanatus*, class I *ClTPSs* contain 17 exons, while most Class II genes contain 3 exons [18]. Despite structural variation, plant TPS proteins typically contain two conserved domains [19]. Functional specialization is observed among TPS subfamilies: Class I TPS genes enhance stress tolerance, whereas Class II TPS genes act as negative regulators of trehalose content. Expression patterns are also tissue-specific and stress-inducible [15]. For example, in tomato, *SlTPP2* and *SlTPP3* are highly expressed in leaves, flowers, and fruits, while *SlTPP8* is predominantly expressed in flowers and green fruits [16]. In sugarcane, *ScTPSs* display differential expression under drought, salt, and ABA treatments [20].

Genome-wide identification and functional characterization of *TPS* genes have been extensively performed in model and crop species such as *Arabidopsis*, rice, and wheat [8,9,10]. However, such a systematic analysis remains lacking in *Morus alba*, a woody plant of economic and ecological importance widely cultivated in China [21]. Among environmental challenges, drought stress critically limits the distribution and productivity of *M. alba*, particularly in arid and semi-arid regions, thereby constraining its industrial and ecological roles [22,23]. Trehalose accumulation can enhance plant growth and development under drought conditions [24,25,26]. Therefore, this study aims to identify the *MaTPS* gene family by genome-wide analysis and investigate their potential roles in drought tolerance through structural characterization, phylogenetic analysis, and expression profiling under drought stress. These analyses provide a comprehensive framework for understanding the structural features, evolutionary relationships, and stress-responsive expression patterns of *MaTPS* genes, thereby contributing to explore their potential involvement in trehalose-mediated drought adaptation in *M. alba*.

## 2. Materials and Methods

### 2.1. Plant Materials

One-year-old seedlings of *Morus alba* L. ‘Baiyu Wang’, obtained from Chengdu, China, were cultivated under field conditions at Chengdu University of Technology, China (104°08′ E, 30°40′ N). Thirty uniform and healthy plants (height: 65 ± 1 cm; basal diameter: 0.225 ± 0.025 cm) were randomly assigned to two groups: a control group (CK) and a drought-stress group (D). Plants in the CK group were irrigated regularly to maintain the soil water content between 50% and 70% relative to field capacity, simulating natural growth conditions. Drought stress was imposed by withholding irrigation in the D group. After 15 days of drought, plants in the D group were rewatered, and rehydration data were recorded on day 20 of the stress treatment.

Mature leaves, stems, and roots were collected from three randomly selected plants per group on days 0, 5, 10, and 15 of the drought treatment, and again on day 5 after rewatering (day 20). All samples were immediately frozen in liquid nitrogen and stored at −80 °C for subsequent analyses.

### 2.2. Trehalose Content Determination

Trehalose content was determined using a trehalose assay kit (A149-1-1; Jiancheng, Nanjing, China) according to the kit protocol. Samples included leaves, stems, and roots from CK and D groups, as well as leaves collected on days 0, 5, 10, and 15 of drought stress and after rewatering (RW). Plant tissues were ground in liquid nitrogen, and 0.05 g of powder was transferred to a centrifuge tube. Extraction was performed by adding 0.5 mL of extraction buffer and homogenizing on an ice-water bath. Samples were incubated at room temperature for 45 min with gentle shaking 3–5 times, then centrifuged at 8000× *g* for 10 min at 25 °C. After collecting the supernatant, blanks, standards, and samples were measured following the kit protocol. Each sample was analyzed in three biological replicates. Absorbance was measured at 620 nm using a spectrophotometer. Trehalose content was calculated as follows:

$$Trehalose\;content\left(mg/mL\right)=\frac{A_{sample}-A_{black}}{A_{standard}-A_{black}}\times C_{standard}\times V_{sample}÷W\times N$$

where A_black_, A_standard_, and A_sample_ are the OD values of the blank, standard, and sample, respectively; C_standard_ is the standard concentration (0.04 μg/mL); V_sample_ is the total extract volume; W is the tissue weight (0.05 g); and N is the dilution factor of the sample before measurement.

### 2.3. MaTPS Gene Family Members Identification

The Pfam seed files containing the conserved protein domains (GT20/PF00982 and TPP/PF02358) of TPS were downloaded from the Pfam database (http://pfam-legacy.xfam.org/, accessed on 12 March 2024). These files were used as the source for identifying *MaTPSs*. The *MaTPS* gene family sequences were acquired from the NCBI (https://www.ncbi.nlm.nih.gov/, accessed on 12 March 2024). *MaTPS* gene family amino acid sequences were compared using BLASTP (v2.12.0, NCBI, Bethesda, MD, USA) to whole genome sequence and eligible sequences were selected. These selected sequences were further analyzed through HMMER v3.3.2 (http://hmmer.org/, accessed on 14 March 2024) searches to obtain the final target gene sequences. Redundant sequences and sequences without *TPS* domains were removed using Blast (https://blast.ncbi.nlm.nih.gov/Blast.cgi, accessed on 16 March 2024) [12].

### 2.4. Phylogenetic Tree Construction of MaTPS Gene Family

TPS protein sequences of *M. alba*, *Arabidopsis thaliana*, and *Populus trichocarpa* were analyzed by multiple sequence alignment using ClustalX v2.1 (EMBL, Heidelberg, Germany). In MEGA v7.0 (Kumar et al., Arizona State University, Tempe, AZ, USA), using the Poisson correction model and 1000 bootstrap replicates, a phylogenetic tree was constructed by the Neighbor-Joining (NJ) method. The visualization and optimization of the phylogenetic tree were performed using TBtools v2.027 (Chen et al., South China Agricultural University, Guangzhou, China).

### 2.5. Gene Features Analysis and Subcellular Localization of MaTPSs

Tbtools and ExPASy (https://www.expasy.org/, accessed on 21 September 2024) were employed to obtain the coding sequences (CDS), gene IDs, chromosomal locations, gene lengths, and subsequently physicochemical properties of *MaTPSs*. Structural and functional analyses were carried out using online tools: transmembrane structure was predicted by TMHMM; signal peptide was identified by SignalP (https://services.healthtech.dtu.dk/services/SignalP-6.0/, accessed on 19 September 2024) [27]; Structural modeling of the protein was conducted using Phyre2 website (https://www.sbg.bio.ic.ac.uk/phyre2/html/page.cgi?id=index, accessed on 2 November 2024); subcellular localization analysis was completed by Plant-mPLoc (http://www.csbio.sjtu.edu.cn/bioinf/plant-multi/, accessed on 13 November 2024); and phosphorylation site of MaTPS proteins was predicted by NetPhos Server (https://services.healthtech.dtu.dk/services/NetPhos-3.1/, accessed on 16 November 2024) [28]. These analyses systematically characterized the *MaTPS* gene family.

### 2.6. Gene Structures and Cis-Acting Elements Analysis of MaTPSs

*MaTPSs* structures were predicted by the Gene Structure Display Server (GSDS 2.0, https://gsds.gao-lab.org/Gsds_help.php, accessed on 16 November 2024) [29]. Based on the genome sequence, the sequences 2000 bp upstream of the ATG start codon of the *MaTPSs* was extracted as a candidate promoter region. Then, promoter cis-acting elements were predicted by PlantCARE online platform (http://bioinformatics.psb.ugent.be/webtools/plantcare/html/, accessed on 16 November 2024) [28,30], and prediction results were visualized and mapped by TBtools to systematically analyze *MaTPS* gene family regulatory features [31].

### 2.7. Protein Conserved Motifs, Domains and Collinearity Analysis of MaTPSs

Conserved structural motifs of *MaTPSs* were identified via MEME online website (https://meme-suite.org/meme/tools/meme, accessed on 17 November 2024). To further examine conserved structural domains of *MaTPSs*, TPS protein sequences were analyzed using online Conserved Domain Database (CDD) provided by the NCBI (https://www.ncbi.nlm.nih.gov/cdd, accessed on 17 November 2024). The collinearity analysis of *TPS* gene family members was performed via MCScanX function in TBtools. This analysis explored the homology of *TPS* gene family members among *M. alba*, *Arabidopsis*, and *Populus*.

### 2.8. MaTPSs Expression Patterns in Leaf Tissues Under Different Treatments

Total RNA was isolated from *M. alba* leaves using the RNAprep Pure Plant Kit (DP441, Tiangen, Beijing, China). RNA concentration and purity were measured using a spectrophotometer (NanoDrop 2000, Thermo Fisher Scientific, Waltham, MA, USA), and RNA integrity was assessed using a Bioanalyzer (Agilent 2100, Agilent Technologies, Santa Clara, CA, USA). FPKM values of *MaTPSs* in drought-stressed leaf tissues were obtained from 10 days unpublished transcriptome data, and the normal watering group was used as the control (Table S4). The values were normalized using TBtools, and a heatmap of expression patterns was generated.

First-strand cDNA was prepared from total RNA with gDNA removed according to the instructions of the FastKing RT kit (KR116, Tiangen, Beijing, China). The actin gene (*M. alba_G0016411*) was used as the internal reference because it exhibited stable expression under the experimental conditions. Specific primers for *MaTPSs* and actin gene were designed following quantitative real-time PCR (qRT-PCR) primer design principles with IDT PrimerQuest (https://sg.idtdna.com/Primerquest/Home/Index, accessed on 23 March 2025). All primer sequences are listed in Table 1. Each 10 µL reaction system consisted of 1 µL of cDNA, 0.2 µL of the forward primer, 0.2 µL of the reverse primer, 5 µL qPCR Master Mix (Lanyun, Beijing, China), and 3.2 µL RNase-free H_2_O. The reaction parameters of qPCR were 94 °C for 20 s, 40 cycles of 94 °C for 10 s and 56 °C for 20 s, and 56 °C for 60 s. The positive control used a known template containing the target gene, while the negative control included a no-template control (NTC) to detect any potential contamination or nonspecific amplification. All qPCR experiments were performed in three replications. Amplification curves were recorded for all samples following qPCR. Cycle threshold (Ct) values were subsequently determined. The 2^−ΔΔCT^ method was employed to calculate relative expression of differentially expressed *MaTPS* genes (DEGs).

### 2.9. Statistical Analysis

All data were organized in Microsoft Excel and analyzed using SPSS 22.0 (SPSS Inc., Chicago, IL, USA). Data are presented as the mean ± standard deviation (SD) of three biological replicates. Prior to analysis of variance, data were tested for normality (Shapiro–Wilk test) and homogeneity of variance (Levene’s test). Differences among treatment means were evaluated by one-way or two-way ANOVA, as appropriate, followed by Tukey’s multiple comparison test. Different lowercase letters indicate significant differences at *p* < 0.05. For correlation analysis, single and double asterisks indicate significant correlations at *p* < 0.05 and *p* < 0.01, respectively [32].

## 3. Results

### 3.1. Trehalose Content in Different Tissues and Drought Periods of M. alba

**Figure 1:**
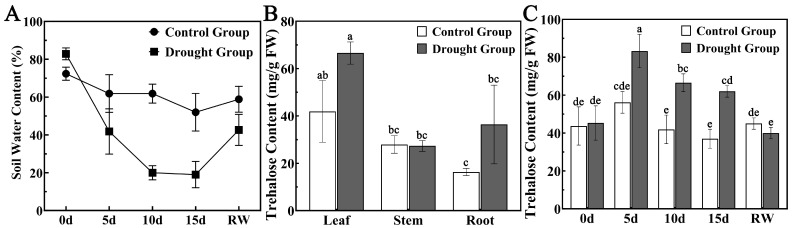
Trehalose content and soil water content in *M. alba* under drought stress. (**A**) Soil water content. (**B**) Trehalose content in different tissues of *M. alba*. (**C**) Trehalose content in leaves under drought stress. Different lowercase letters (a, b, c, d, e) indicate significant differences at *p* < 0.05.

Under drought stress, the soil water content in the control group remained between 50% and 70%. On day 5 of drought stress, the soil water content decreased to approximately 40%, and on days 10 and 15, it remained at 20% (Figure 1A). Significant variation in trehalose content was observed across leaf, stem, and root tissues under drought stress. Drought-stressed *M. alba* plants exhibited significantly higher trehalose concentrations than the control group, with a 1.6-fold increase in leaf and a 2.2-fold increase in root tissue (Figure 1B). No significant changes were observed in stem tissue under drought stress. In leaf tissue, trehalose content increased continuously with drought stress duration, peaking at day 5 (1.5-fold), and then gradually decreasing (Figure 1C).

### 3.2. Identification and Chromosomal Location of MaTPSs

TPS is a key enzyme that catalyzes trehalose biosynthesis. All 11 *MaTPS* members were contain both PF00982 and PF02358 domains. Their lengths differ widely, and *MaTPS5* is the longest at 24,333 bp, while *MaTPS1* is the shortest at 3915 bp (Table S1). Physicochemical properties analysis of MaTPS proteins was performed, including isoelectric points (pI, 5.58 to 6.66) and molecular weights (MW, 96.146 to 105.357 kDa) (Table 2). Grand average of hydropathicity (GRAVY) of MaTPS proteins was −0.208, suggesting that all are hydrophilic proteins (Figure S1). They lack transmembrane domains and contain no signal peptides (Figure S2). Phosphorylation is a key regulatory mechanism for protein activity and stability in plants. Our analysis revealed greater sequence diversity in the N- and C-terminal regions of MaTPS proteins compared to the conserved central domain. Multiple phosphorylation sites, including serine, threonine, and tyrosine residues, were identified (Table S2).

**Figure 2:**
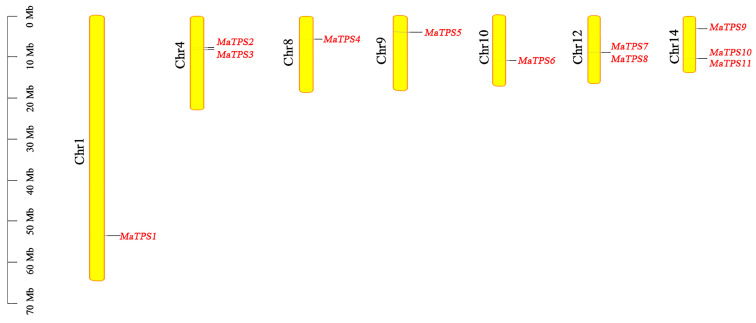
Chromosomal localization of *MaTPSs*.

Subcellular localization prediction indicated that most *MaTPSs* were predicted to localize to vacuole and chloroplast, whereas *MaTPS2* localized to cytoplasm (Table S3). These localization patterns suggest functional diversification among the *MaTPS* family members. Additionally, MaTPS proteins share conserved structural architectures, with particularly close superimposition observed between *MaTPS7*/*MaTPS8* and *MaTPS10*/*MaTPS11* (Figure S3). *MaTPSs* are distributed on chromosomes Chr1 (*MaTPS1*), Chr4 (*MaTPS2,3*), Chr8 (*MaTPS4*), Chr9 (*MaTPS5*), Chr10 (*MaTPS6*), Chr12 (*MaTPS7,8*), and Chr14 (*MaTPS9,10,11*) according to the *M. alba* genome information (Figure 2).

### 3.3. MaTPS Gene Family Phylogenetic Analysis

**Figure 3:**
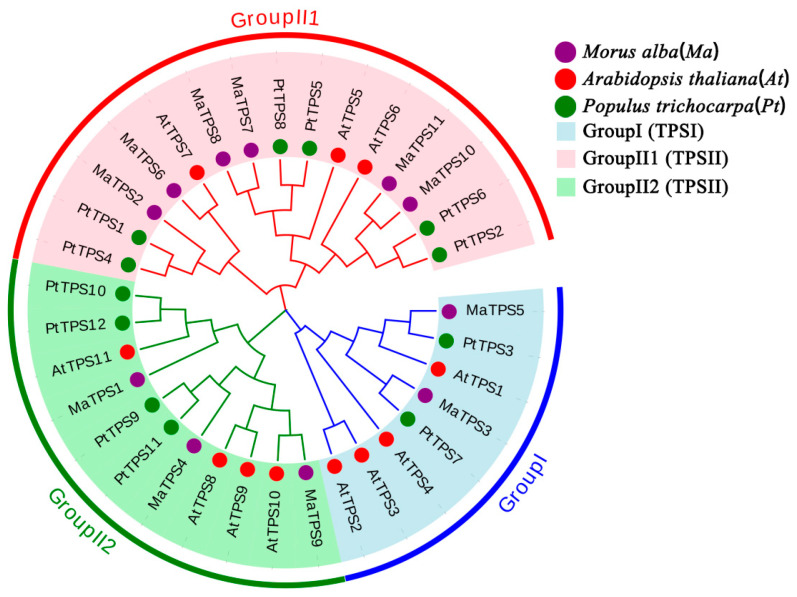
Phylogenetic trees of *MaTPSs*, *AtTPSs*, and *PtTPSs*. The tree is divided into three main groups: Group I (blue region), Group II1 (pink region), and Group II2 (green region). The purple, red, and green circles represent *MaTPSs*, *AtTPSs*, and *PtTPSs*, respectively.

Multiple sequence alignments were performed for *AtTPSs, PtTPSs* and *MaTPSs* to characterize *MaTPSs* family evolutionary relationships. 34 *TPSs* could be classified into TPS I (Group I) and TPS II (Group II1 and Group II2) subfamilies (Figure 3). Specifically, TPS I included four *AtTPSs*, two *PtTPSs*, and two *MaTPSs*. TPS II consisted of seven *AtTPSs*, ten *PtTPSs*, and nine *MaTPSs*. TPS II was dominant subfamily with nine *MaTPSs*; TPS I contained only *MaTPS3* and *MaTPS5*. The phylogenetic tree shows that most *MaTPSs* cluster with *PtTPSs* in the same branch, while they are more distantly related to *AtTPSs*.

### 3.4. Gene Structures and Cis-Acting Elements Analyses of MaTPSs

**Figure 4:**
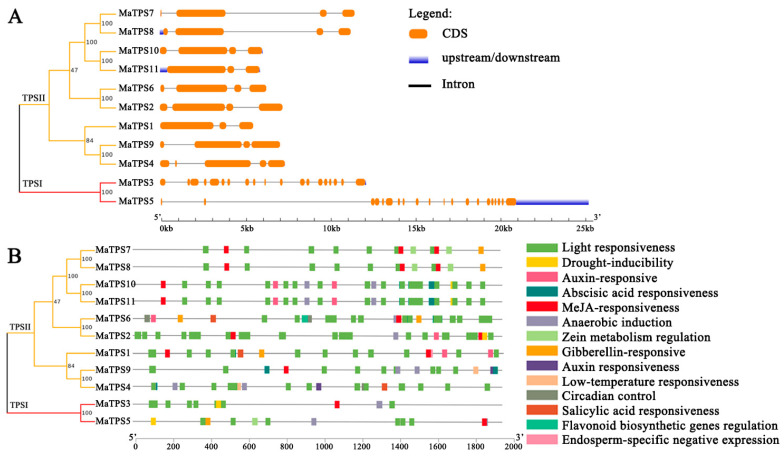
Gene structures and cis-acting elements of *MaTPSs*. (**A**) Organization of exons and introns in *MaTPSs*. Orange rounded rectangles denote exons, blue rectangles signify untranslated regions (UTRs), and black lines indicate introns. (**B**) Cis-acting elements in *MaTPSs* promoters. Distinct colored boxes represent different functional elements, such as light responsiveness (green), drought inducibility (yellow), auxin-responsive (pink), and so on.

Analysis of intron-exon organization in *MaTPSs* revealed significant differences between TPS I and TPS II subfamilies, and members of same subfamily exhibited conserved gene structures in *M. alba* (Figure 4A). Most *MaTPSs* contain 4 exons, while *MaTPS3* and *MaTPS5* contain 19 and 20 exons, respectively. 14 types of cis-acting elements were identified in the promoters of *MaTPSs*, suggesting their involvement in multiple regulatory pathways, including stress responses, phytohormone signaling, circadian regulation, and growth and differentiation (Figure 4B). Notably, *MaTPS4* and *MaTPS9* contain low-temperature responsive cis-elements, while *MaTPS10* and *MaTPS11* have the most hormone-responsive elements.

### 3.5. Conserved Motifs, Structural Domains, and Multiple Sequence Alignment Analysis of MaTPSs

**Figure 5:**
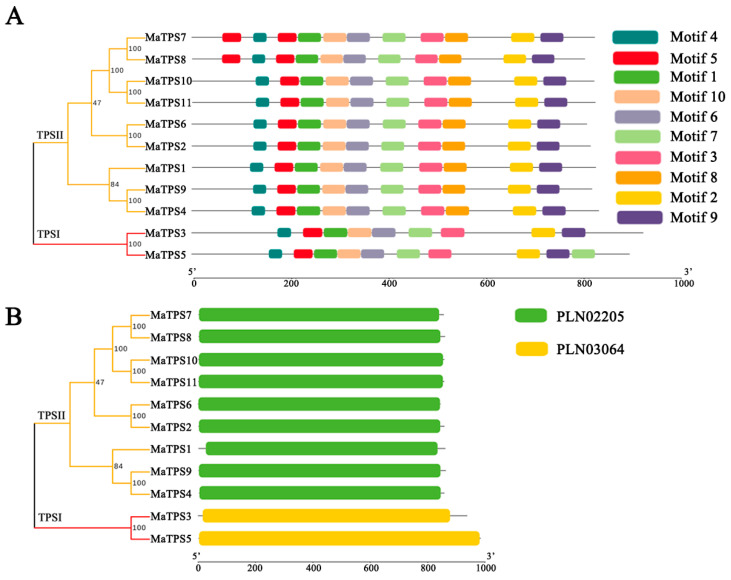
Motif distribution and conserved protein domains of *MaTPSs*. (**A**) Motif composition of MaTPS proteins. Distinct colored boxes are used to depict the 10 conserved motifs. (**B**) Protein domain architecture.

Ten conserved motifs, named Motif 1 to Motif 10, were identified in the MaTPS proteins, with lengths ranging from 29 to 50 amino acids (Figure S4). TPS II subfamily members exhibit similar motif arrangements, indicating their evolutionary conservation. In contrast, TPS I subfamily members lack motif 8 compared to TPS II, which may reflect functional differences between the two subfamilies (Figure 5A). MaTPS proteins contain a single structural domain, with TPS I subfamily members harboring the PLN03064 domain and TPS II subfamily members the PLN02205 domain, both belonging to the UDP-forming domain family (Figure 5B). Multiple sequence alignment of *MaTPSs* revealed that *MaTPS7*/*MaTPS8* and *MaTPS10*/*MaTPS11* pairs have complete sequence identity, and *MaTPS3* and *MaTPS5* shared 78.26% similarity (Figure S5).

### 3.6. Collinearity Analysis of MaTPSs

**Figure 6:**
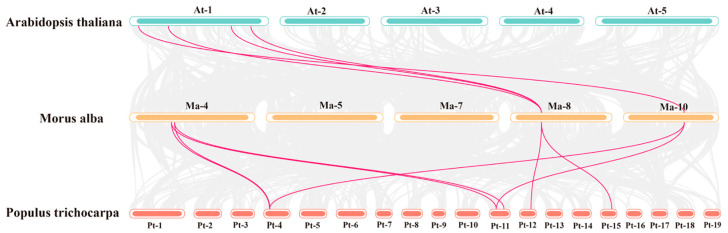
Collinearity Analysis of *MaTPSs* between *Arabidopsis*, *Populus* and *M. alba*.

To explore the evolutionary conservation of the *MaTPS* gene family in *M. alba*, we performed collinearity analysis by comparing it with the herbaceous model plant *Arabidopsis thaliana* and the woody model plant *Populus* (Figure 6). Consistent with the phylogenetic relationships, *M. alba* is more closely related to *Populus*, as indicated by a greater number of collinear gene pairs. Specifically, 4 pairs of collinear genes were identified between 11 *MaTPSs* and 11 *AtTPSs*, while 8 pairs of collinear genes were detected between 11 *MaTPSs* and 12 *PtTPSs*.

### 3.7. Analysis of MaTPSs Expression Profiles in Leaves

**Figure 7:**
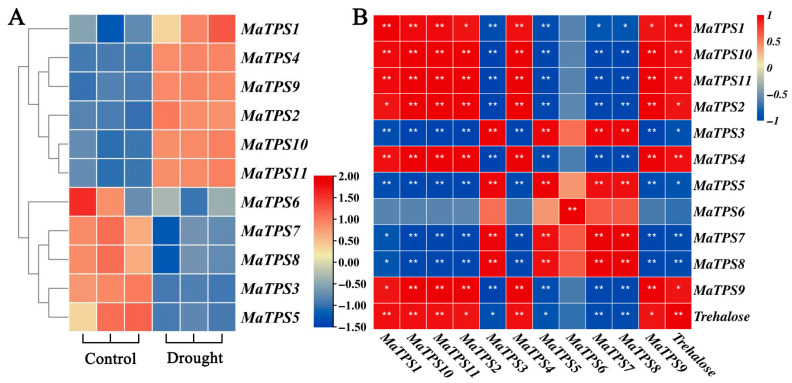
Expression patterns of *MaTPS* genes in leaves under drought stress and their correlations with trehalose content. (**A**) Heatmap of the expression patterns of 11 *MaTPS* genes. (**B**) Correlation between trehalose content and the expression of 11 *MaTPS* genes. * and ** indicate significant correlations at *p* < 0.05 and *p* < 0.01, respectively.

To explore *MaTPSs* functions under drought stress, expression patterns of *MaTPSs* in leaf tissues were analyzed (Figure 7A). *MaTPS1*, *MaTPS2*, *MaTPS4*, *MaTPS9*, *MaTPS10*, and *MaTPS11* were upregulated under drought stress, with *MaTPS4* showing the most prominent induction. In contrast, *MaTPS3*, *5*, *6*, *7*, and *8* were downregulated on day 10 of drought stress, potentially participating in the dynamic regulation of stress responses. *MaTPS7*/*8* and *MaTPS10*/*11* exhibited similar expression change trends under both control and drought stress conditions. Such co-expressed gene pairs may indicate the existence of a coregulatory relationship within the gene regulatory network.

To explore the relationship between trehalose accumulation and *MaTPS* gene expression, correlation analysis was performed between trehalose content and the expression levels of 11 *MaTPS* genes (Figure 7B). Trehalose content was significantly positively correlated with the expression of *MaTPS1*, *MaTPS2*, *MaTPS4*, *MaTPS9*, *MaTPS10*, and *MaTPS11* (*p* < 0.05), with correlation coefficients all greater than 0.90. Particularly strong positive correlations were observed for *MaTPS1*, *MaTPS4*, *MaTPS10*, and *MaTPS11* (*p* < 0.01). In contrast, trehalose content was significantly negatively correlated with the expression of *MaTPS3*, *MaTPS5*, *MaTPS7*, and *MaTPS8* (*p* < 0.05), with particularly strong negative correlations for *MaTPS7* and *MaTPS8* (*p* < 0.01).

### 3.8. Analysis of MaTPSs Expression Patterns Under Drought Stress

**Figure 8:**
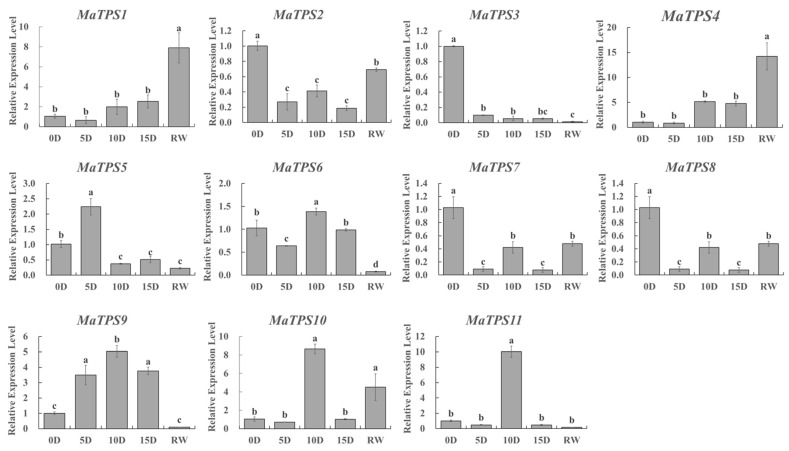
Expression patterns of *MaTPSs* in leaf tissues under drought stress. Different lowercase letters (a, b, c, d) indicate significant differences at *p* < 0.05.

The role of *MaTPSs* during drought tolerance was investigated by qRT-PCR analysis of 11 *MaTPSs* expression profiles (Figure 8). Results indicated that the expression levels of TPS I member *MaTPS3* and *MaTPS5* were significantly downregulated under drought stress, with expression levels decreasing by 11.12-fold and 1.82-fold, respectively, on day 10 of drought. Most TPS II members showed an expression level change that first rose and then fell during drought stress. Specifically, expression levels of *MaTPS4*, *MaTPS6*, *MaTPS9*, *MaTPS10*, and *MaTPS11* peaked on day 10, reaching 5.15-, 1.39-, 5.04-, 8.65-, and 10.02-fold compared to the control, respectively. *MaTPS1* showed the highest expression level on day 15, which was 2.53-fold higher than control. Conversely, expression levels of *MaTPS2*, *MaTPS7*, and *MaTPS8* were reduced to two-fifths of control levels on the 10th day of drought. Following rehydration, only *MaTPS1*, *MaTPS4*, and *MaTPS10* recovered to control levels or higher.

## 4. Discussion

### 4.1. Trehalose Accumulation and Drought Response of MaTPS Genes in Morus alba

Trehalose is increasingly recognized as a critical metabolite that enhances plant tolerance to abiotic stress [24]. Under drought conditions, trehalose contributes to osmotic adjustment and stabilization of cellular structures, thereby protecting plants from dehydration-induced damage [26]. However, most plants maintain extremely low endogenous levels of trehalose, which are generally insufficient to confer effective osmotic protection [33]. In such cases, exogenous trehalose application has been shown to modulate metabolism and improve stress tolerance, as demonstrated in *Rosa rugosa* [34].

In the present study, trehalose accumulated significantly in the leaves and roots of *M. alba* under drought stress (27.324 to 66.557 mg/g FW), with levels markedly higher than those reported in *Arabidopsis* (37 ng/g DW) and transgenic *Oryza sativa* (1.076 mg/g FW) [35]. This substantial accumulation suggests that *M. alba* may possess an efficient trehalose-mediated drought adaptation mechanism. In addition to its osmoprotective role, trehalose metabolism is closely associated with signaling pathways, particularly through trehalose-6-phosphate (Tre6P), which functions as a key regulator of carbon metabolism and stress responses. Therefore, the observed increase in trehalose likely reflects both metabolic adjustment and broader regulatory responses to drought stress.

Consistent with trehalose accumulation, several *MaTPS* genes (*MaTPS1*, *MaTPS2*, *MaTPS4*, *MaTPS9*, *MaTPS10*, and *MaTPS11*) were upregulated under drought conditions, suggesting their involvement in trehalose biosynthesis (Figure 7). In contrast, *MaTPS3*, *MaTPS5*, *MaTPS7*, and *MaTPS8* were downregulated, indicating functional differentiation within the *TPS* gene family. Variations in exon–intron organization were observed between TPS I and TPS II subfamilies, as well as within the TPS II group. Similar patterns of structural divergence have been reported in sugarcane and peach, suggesting that functional diversification of *TPS* genes is widespread in plants [15,20,36]. Notably, all significantly upregulated *MaTPS* genes in this study belonged to the TPS II subfamily, consistent with findings in *Chenopodium quinoa*, further supporting their potential regulatory roles in stress adaptation [7].

### 4.2. Evolutionary Conservation and Regulatory Complexity of the TPS Gene Family

Phylogenetic analysis revealed that *TPS* gene family members are generally conserved within taxonomic groups. For instance, leguminous species such as *Arachis hypogaea* and *Medicago truncatula* cluster closely with *Glycine max*, while *Camphora longepaniculata* groups with its congeners [37,38]. In this study, MaTPS proteins showed close phylogenetic relationships with *TPS* genes from the woody genus *Populus*, indicating evolutionary conservation among woody plants (Figure 3 and Figure 6).

Interestingly, the expression patterns of *TPS* genes in *M. alba* appear to differ from those reported in some herbaceous species. While TPS I genes in species such as *Arabidopsis* and maize are often upregulated under stress conditions, their expression in woody plants may be more tightly regulated [8,39,40]. This suggests that perennial species may adopt distinct regulatory strategies to maintain metabolic balance and avoid excessive accumulation of intermediates such as Tre6P [41].

Subcellular localization analysis further demonstrated that MaTPS proteins are distributed across chloroplasts, vacuoles, and the cytoplasm, similar to TPS proteins in *Citrullus lanatus* [18]. This diverse localization implies functional specialization across cellular compartments. In addition, promoter analysis identified multiple cis-acting elements related to drought, light, low temperature, flavonoid biosynthesis, and hormone responses, indicating that *MaTPS* genes are likely involved in complex regulatory networks integrating environmental signals and developmental processes [38,42,43,44].

This study has several limitations, as the functions of *MaTPS* genes were inferred mainly from expression and metabolite data without direct genetic or protein-level validation. Future work should focus on functional characterization of *MaTPS4*/*9*/*10*/*11* candidate genes using gene editing and multi-omics approaches to clarify their roles in drought response. Overall, our results suggest that *MaTPS* genes may participate in drought adaptation through the regulation of trehalose metabolism, providing a basis for future studies on drought tolerance improvement in mulberry.

## 5. Conclusions

In this study, genome-wide identification and systematic analysis of the *TPS* gene family were performed in *M. alba*. A total of 11 *MaTPS* genes were identified and classified into two subfamilies, TPS I and TPS II. Analyses of gene structure, conserved motifs, phylogenetic relationships, and cis-acting regulatory elements indicated that the *MaTPS* gene family has maintained a certain degree of evolutionary conservation while also exhibiting considerable regulatory complexity. Drought stress significantly promoted trehalose accumulation and altered the expression of multiple *MaTPS* genes. Among them, *MaTPS1*, *MaTPS2*, *MaTPS4*, *MaTPS9*, *MaTPS10*, and *MaTPS11* were upregulated, whereas *MaTPS3*, *MaTPS5*, *MaTPS7*, and *MaTPS8* were downregulated, suggesting functional diversification within this gene family. Correlation analysis further indicated that trehalose accumulation was significantly positively correlated with the upregulated *MaTPS* genes and negatively correlated with several downregulated genes. Overall, this study provides a foundation for elucidating the potential roles of *MaTPS* genes in the drought response of *M. alba* and offers candidate gene resources for further functional validation and the genetic improvement of drought tolerance in mulberry.

## Figures and Tables

**Table 1:** Primer sequences for qRT-PCR experiments.

Gene	Forward Primer Sequence	Reverse Primer Sequence
*MaActin* (*M. alba_G0016411*)	AAGATGCTCTACGCCACATC	CCCGTTCTCTAAGCACTTCAA
*MaTPS1*	GAGCAGAAAGAAAGTGCATTGG	CACTCTCAAGGTGGTCAAGAAG
*MaTPS2*	CTGGATCGCCAAGTTCTTCT	CACTTCCAGACACGGCTAAA
*MaTPS3*	CTGCTGATTTGGTTGGTTTCC	CAGCTTTCCTTGATCCTCTACTC
*MaTPS4*	GATGTGCCTGGACCATTGTA	TCACCTCCACGACCTTATCT
*MaTPS5*	AGGCGGAAATAAGGACAAGAG	GTTGCGTTGAATCCCAGTATAAG
*MaTPS6*	TTTATTGGGTGCTCTCCATCC	CTCTTGCTCTGAATCCCTCATC
*MaTPS7*	CTATGGATTCTGCCCTTGTAGTC	AGAAACTCCTCGCCCAATATG
*MaTPS8*	CTATGGATTCTGCCCTTGTAGTC	AGAAACTCCTCGCCCAATATG
*MaTPS9*	TCGGAATCTCGCCCAAATC	TCGGAATCTCGCCCAAATC
*MaTPS10*	ACGAGATGTGGAATGGGAAAC	CCGTCTGTTGCTTCTGTGTAA
*MaTPS11*	AGTCGCCAAGGGAATGATAAG	CAGCCAATAAACTCGGAGACA

**Table 2:** Physicochemical Properties of MaTPS Proteins.

Gene Name	Isoelectric Points	Molecular Weights (Da)	Protein Length (AA)	Instability Index	Aliphatic Index	Hydrophilicity Index
*MaTPS1*	5.66	96,719.47	858	50.28	86.78	−0.212
*MaTPS2*	5.87	96,214.81	856	50.19	86.55	−0.214
*MaTPS3*	6.59	105,357.46	936	44.67	89.25	−0.29
*MaTPS4*	6.66	96,872.51	854	53.46	90.71	−0.155
*MaTPS5*	6.43	104,729.9	929	42.6	83.42	−0.395
*MaTPS6*	5.58	96,145.65	843	46.62	86.33	−0.273
*MaTPS7*	5.8	96,658.69	857	47.07	94.15	−0.168
*MaTPS8*	5.8	96,658.69	857	47.07	94.15	−0.168
*MaTPS9*	5.92	96,543.87	861	50.49	92.94	−0.141
*MaTPS10*	5.82	96,581.24	855	42.21	91.85	−0.134
*MaTPS11*	5.82	96,581.24	855	42.21	91.85	−0.134

## Data Availability

The original contributions presented in this study are included in the article/Supplementary Materials. Further inquiries can be directed to the corresponding author.

## Supplementary Materials

The following supporting information can be downloaded at https://www.mdpi.com/article/10.3390/cimb48040356/s1.
