# Supplementary material for: Genomic Analysis of the Trehalose-6-Phosphate Synthase Family Involved in Trehalose Biosynthesis and Drought Response in Morus alba
Source: Curr Issues Mol Biol. 2026 Mar 28;48(4):356. doi: 10.3390/cimb48040356 (PMC13115105; doi:10.3390/cimb48040356)
Supplement: Supplementary file 1 [file cimb-48-00356-s001.zip › Supplementary material Figures S1-S5.pdf]

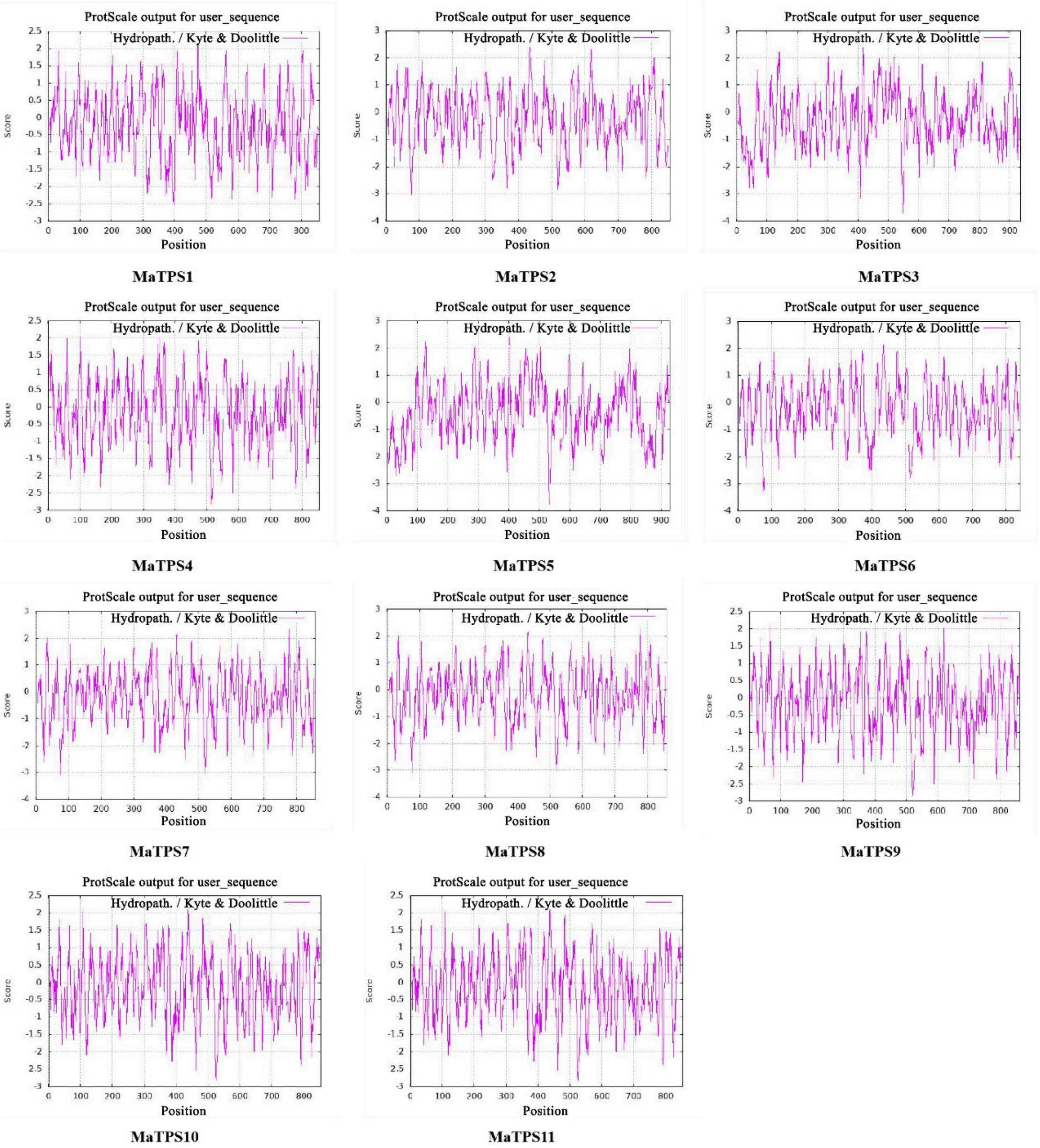

Figure S1. Hydrophilic analysis of *MaTPSs*

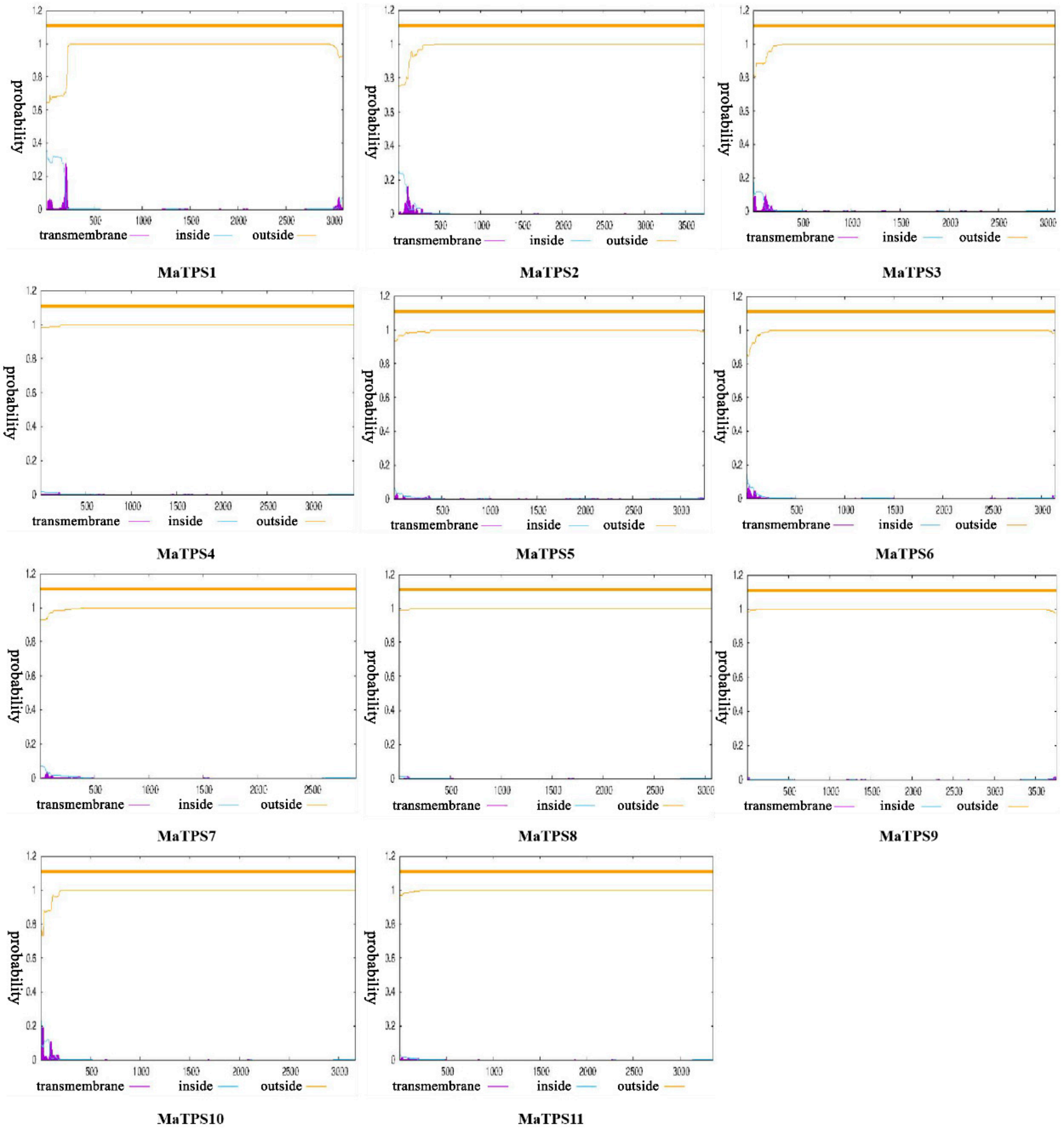

Figure S2. Transmembrane Domain Analysis of *MaTPSs*

Note: purple line: transmembrane; blue line: intracellular; yellow line: extracellular.

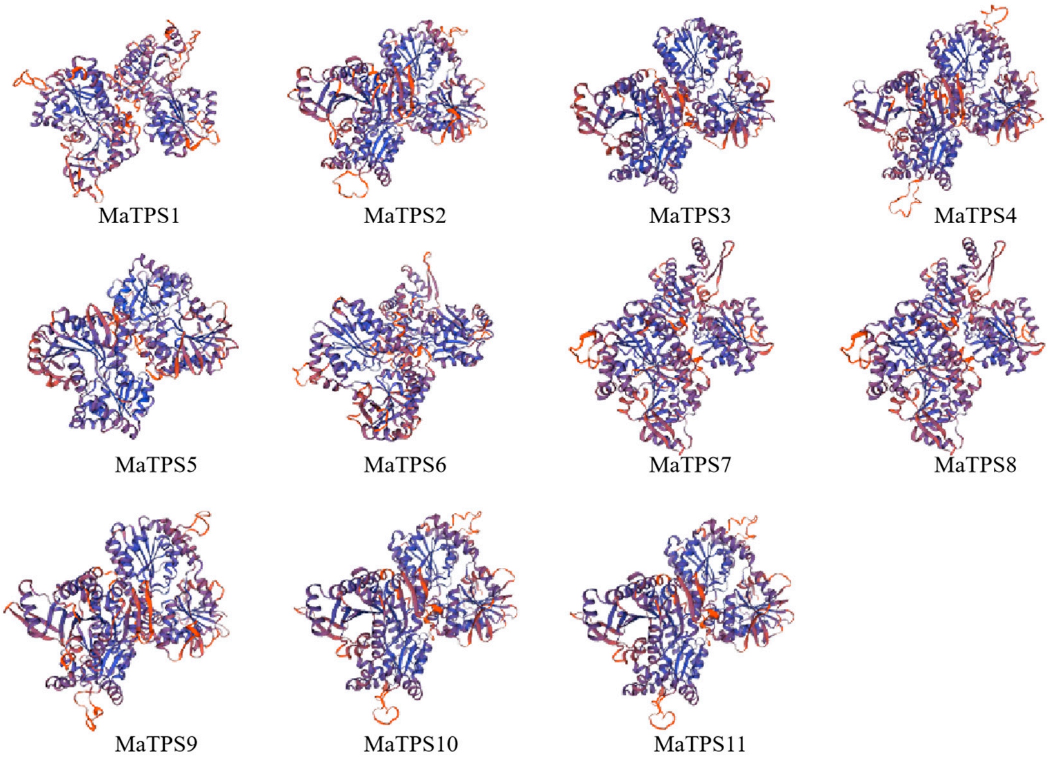

Figure S3. Predicted 3D Structural Models of 11 MaTPS Proteins in *M. alba*

Motif1

Motif2

Motif3

Motif4

Motif5

Motif6

Motif7

Motif8

Motif9

Motif10

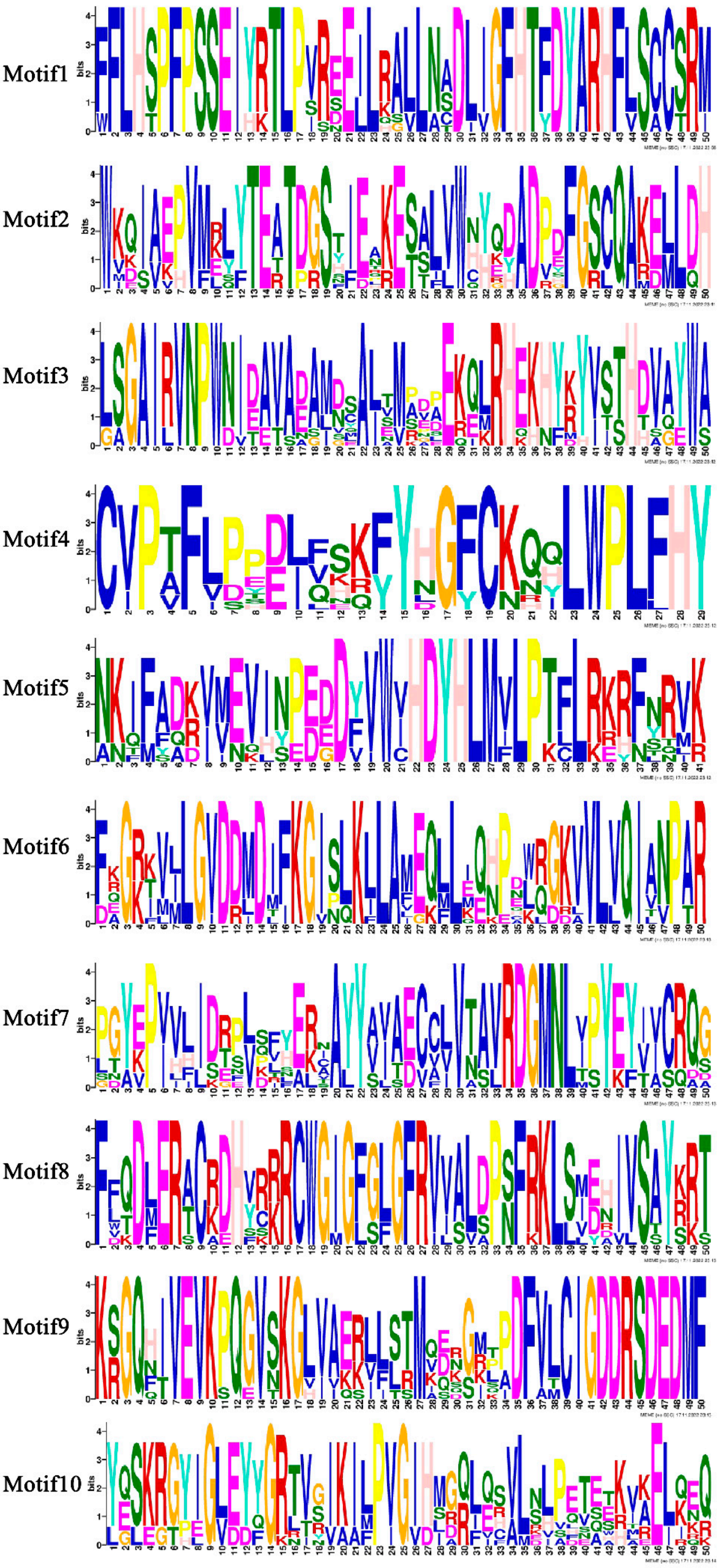

Figure S4. Sequence logos of the 10 conserved motifs of *MaTPSs*

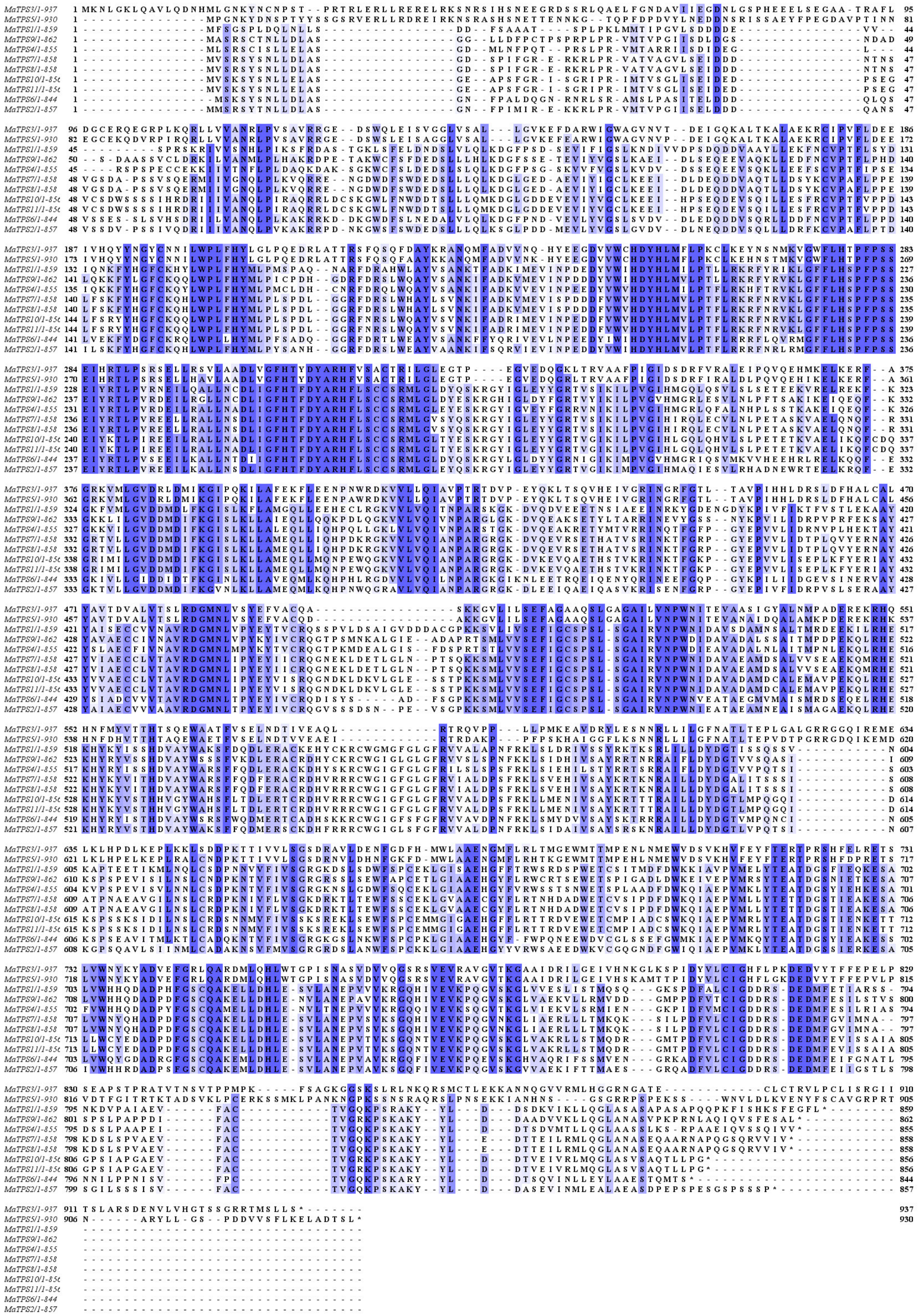

Figure S5. Multiple Sequence Alignment of *MaTPSs* Amino Acid Sequences in *M. alba*

Note: Blue areas indicate consistency≥30 per cent; the higher the consistency, the darker the blue

colour.
